# Factors influencing the outcome after surgical reconstruction of OTA type B and C tibial plateau fractures: how crucial is the restoration of articular congruity?

**DOI:** 10.1007/s00402-022-04405-5

**Published:** 2022-03-18

**Authors:** Thomas Rosteius, Valentin Rausch, Simon Pätzholz, Sebastian Lotzien, Matthias Königshausen, Thomas Armin Schildhauer, Jan Geßmann

**Affiliations:** 1 Department of General and Trauma Surgery, BG University Hospital Bergmannsheil, Bürkle-de-la-Camp Platz 1, 44789 Bochum, Germany; 2 Department of Radiological Diagnostics, Interventional Radiology and Nuclear Medicine, BG University Hospital Bergmannsheil, Bürkle-de-la-Camp Platz 1, 44789 Bochum, Germany

**Keywords:** Tibial plateau fractures, Articular congruity, CT measurement, Outcome

## Abstract

**Introduction:**

Only few and inconsistent data about the impact of articular congruity and tolerable residual intraarticular steps and gaps of the joint surface after tibial plateau fractures exist. Therefore, aim of this study was to investigate the correlation between OTA type B and C tibial plateau fracture outcomes and postoperative articular congruity using computed tomography (CT) data.

**Materials and methods:**

Fifty-five patients with a mean age of 45.5 ± 12.5 years and treated for 27 type B and 28 C tibial plateau fractures with pre- and postsurgical CT data were included. Primary outcome measure was the correlation of postoperative intraarticular step and gap sizes, articular comminution area, the postoperative medial proximal tibial angle (MPTA), and the Lysholm and IKDC score. Receiver-operating characteristic (ROC) curves were used to determine threshold values for step and gap heights according to the following outcome scores: IKDC > 70; Lysholm > 80. Secondary outcome measures were the correlation of fracture severity, the number of complications and surgical revisions and the outcome scores, as well as the Tegner activity score before injury and at final follow-up.

**Results:**

After a mean follow-up of 42.4 ± 18.9 months, the mean Lysholm score was 80.7 ± 13.3, and the mean IKDC score was 62.7 ± 17.6. The median Tegner activity score was 5 before the injury and 4 at final follow-up (*p* < 0.05). The intraarticular step height, gap size, comminution area and MPTA deviation were significantly negatively correlated with the IKDC and Lysholm scores. The cutoff values for step height were 2.6 and 2.9 mm. The gap size threshold was 6.6 mm. In total, an average of 0.5 ± 0.8 (range 0–3) complications occurred, and on average, 0.5 ± 1.1 (range 0–7) surgical revisions had to be performed. The number of complications and surgical revisions also had negative impacts on the outcome. Neither fracture severity nor BMI or patient’s age was significantly correlated with the IKDC or Lysholm score.

**Conclusions:**

Tibial plateau fractures are severe injuries, which lead to a subsequent reduced level of patient activity. Precise reconstruction of the articular surface with regard to intraarticular step and gap size, residual comminution area and joint angle is decisive for the final outcome. Complications and surgical revisions also worsen it.

**Level of evidence:**

III.

## Introduction

Although tibial plateau fractures account for only 1% of all fractures [1, 2] with an incidence of 10.3/100,000/year [3], these fractures are one of the most severe and challenging injuries involving the knee joint. This is partly due to it being an uncommon, complex articular surface injury with very heterogenic fracture morphology and a high rate of accompanying soft tissue injuries, such as compartment syndrome and vascular, chondral, ligament and meniscal lesions [3–6]. Moreover, despite advances in diagnostics and surgical methods, high rates of posttraumatic osteoarthritis up to 40% [7] and large proportions with poor outcomes still remain [8], which could be due to an incongruent restoration after osteosynthesis. This is particular important since optimum restoration of the joint surface and articular congruity seems to be decisive for achieving satisfactory clinical results [7, 9, 10].

Hence, (1) an exact analysis of the fracture using computed tomography (CT)/magnetic resonance imaging (MRI) for diagnosis [5, 11] and (2) preoperative planning of the surgical strategy accounting for the possible need for different approaches are crucial for achieving the desired anatomical reconstruction and clinical result [12–14]. Unfortunately, data regarding the tolerable level of articular incongruity that are needed to achieve an acceptable outcome and a low risk of posttraumatic osteoarthritis are controversial [15]. The postulated articular incongruity values range from nearly 2–10 mm in the case of tibial plateau fractures [7, 9, 15–18]. Furthermore, the detailed impact of intraarticular fracture steps, gaps, bony defects, and axial malalignment remaining postoperatively on functional outcome has poorly been investigated. This is of particular clinical importance since after reconstruction of tibial plateau fractures, orthopedic surgeons are often faced with the question of what level of articular incongruity is to be tolerated or surgically revised.

Therefore, the aim of this study was to analyze the subjective and functional outcomes after complex OTA type B and C tibial plateau fractures in relation to postoperative articular congruity and axial alignment by means of a detailed pre- and postsurgical CT and X-ray analysis. In addition, we aimed to find an appropriate cutoff value for an acceptable functional outcome to give orthopedic surgeons a guideline regarding tolerable joint position and congruity. We hypothesized that (1) more articular congruity leads to better functional outcomes and (2) complications and surgical revisions have a negative impact.

## Materials and methods

The study was reviewed and approved by the local Institutional Review Board (IRB) (registered number: 18-6508_1-BR). All procedures were performed in accordance with the ethical standards of the institutional research committee and with the 1964 Declaration of Helsinki and its later amendments.

### Study design

Patients with tibial plateau fracture between 08/2013 and 03/2018 were prospectively reviewed. All patients with OTA type B or C fractures and a minimum follow-up of 1 year were included in this study. Exclusion criteria were residual ligamentous joint instability and missing pre- and postsurgical CT data. Moreover, patients with accompanying collateral ligament or meniscal injury, vascular damage or previous damage of the joint were excluded from further analysis. In total, 68 patients fulfilled these criteria. Fifty-five out of these 68 (80.9%) patients were available for clinical follow-up and were considered for further analysis.

### Surgical management and postoperative procedures

In all cases, the fractures were stabilized using internal plating via standard anterolateral/ anteromedial and posteromedial approaches. All OTA type B fractures were fixed using a medial/lateral single locking plate, whereas type C fractures were fixed using double plating.

Lower leg radiographs and CT images were obtained during the first 72 h after surgery to analyze the axial alignment and postsurgical articular congruity.

Physical therapy started 48 h after the operation with passive motion of the joint through a limited range of motion (ex./flex. 0°/0°/90°) with the patient in the supine position. If necessary, peripheral nerve block anesthesia was applied. Patients had limited weight bearing (20 kg) and limited range of motion for 6 weeks.

### Follow-up examination

The patient assessment and clinical evaluation were scheduled a minimum of 1 year after the primary surgery. Subjective and functional outcomes were determined using the Lysholm and Tegner activity scores [19] and the International Knee Documentation Committee (IKDC) subjective knee form score [20]. The range of motion (ROM) was measured using a goniometer.

### Measurement of the postsurgical CT scans and radiographs

The postoperative congruity of the articular surface of the tibial plateau was measured using postoperative CT. Therefore, multiplanar reformations (MPR) of the knee joint dataset in coronal, sagittal and axial sections were used. Reconstructions had a layer thickness of 0.75 mm. A B60s kernel was used for image reconstruction; the measurement was made in the bone window (W2000:L500). Measurement of the tibial plateau was defined 3 mm below the articular surface on axial CT sections. Coronary and sagittal CT sections were normalized to the anatomical axis of the tibia.

We analyzed the number of intraarticular fragments without bony connection to the tibial metaphysis, the intraarticular step height and gap size in mm, and the size of a remaining comminution area measured in mm^2^. The intraarticular steps and gaps were determined using sagittal, axial and coronal CT sections, whereas the comminution area of the tibial plateau was measured on axial CT sections. The maximum extent of the step, gap and comminution area was always specified.

**Fig. 1:**
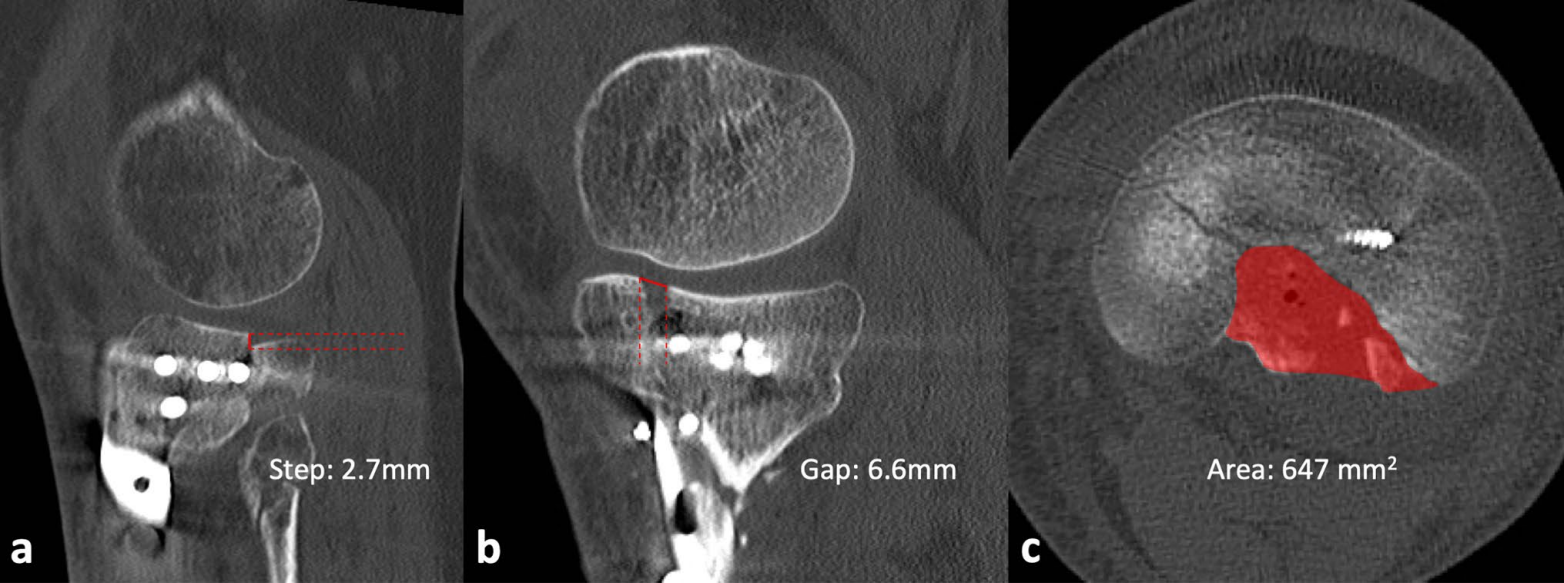
Digital measurement of steps, gaps and comminution area of the articular surface on CT scans. Examples of maximum remaining step height (**a**), gap size (**b**) and size of the comminution area of the articular surface (**c**) on postoperative CT scans after surgical reconstruction of the tibial plateau fractures

Axial alignment based on the deviation of the medial proximal tibia angle (MPTA) according to Paley [21] was determined on the postsurgical lower leg X-ray and compared to the opposite side or a normative standard. The measurement was carried out using the digital measurement tool of our PACS client (IMPAX, Agfa HealthCare GmbH, Bonn, Germany). Figure 1 demonstrates digital measurement of the articular surface.

### Statistical analysis

Descriptive data are described by the mean, median, standard deviation, minimum and maximum. After testing for normal distribution of data using the Shapiro–Wilk test, significance was calculated using a two-tailed *t* test or Wilcoxon/Mann–Whitney test. To demonstrate a correlation, we calculated Spearman’s rank correlation coefficient. According to a power analysis carried out, a sample size of 46 patients was required for a significant result. To determine appropriate cutoff values of intraarticular steps and gaps for predicting a successful outcome, we used receiver-operating characteristic (ROC) curves. The minimum outcome value of the Lysholm score was set to 80, and the minimum IKDC value was set to 70. Data were processed in SPSS statistics (IBM, Armonk, USA). *α* = 0.05 or less was considered statistically significant.

## Results

In total, 27 (49.1%) patients had an OTA type B fracture, while 28 (50.9%) patients were diagnosed with a type C fracture. Twenty-four patients (43.6%) were female. Table 1 shows the demographic data and concomitant injuries of the study group.

Table 2 demonstrates the final outcome and complications after a mean follow-up of 42.4 ± 18.9 months (range 12–75 months). There were no statistically significant differences between OTA type B and C fractures in terms of the Lysholm and IKDC scores (*p* = 0.340; *p* = 0.274). Patients with at least one postsurgical complication had significantly lower IKDC scores (56.2 vs. 66.4; *p* = 0.037). Patients with a bony surgical revision also had lower Lysholm (72.7 vs. 82.3; *p* = 0.064) and IKDC (48.5 vs. 65.4; *p* = 0.007) scores. Both the number of complications (rho − 0.4; *p* = 0.003/rho − 0.3; *p* = 0.036) and the number of surgical revisions (rho − 0.5; *p* = 0.000/rho − 0.4; *p* = 0.003) also correlated significantly negatively with the outcome. The Tegner Activity Score was significantly lower at final follow-up (median 4) compared to the pre-injury level (median 5) (*p* < 0.001).

The postsurgical X-ray and CT analysis of the axis and fracture morphology is demonstrated in Table 3. In all cases with an existing residual intraarticular step, the step remains due to an undercorrection/ residual depression with regard to the joint line (Fig. 1a). The intraarticular step height level, the size of the gap, the size of the comminution area and the deviation of the MPTA correlated significantly with low IKDC and Lysholm scores. Table 4 shows the individual Spearman rank correlations between these measurements and the outcome scores.

There were no significant correlations between the number of fracture fragments, the patient’s age or BMI with either the IKDC (rho − 0.189; *p* = 0.166), (rho − 0.096; *p* = 0.486) (rho − 0.03; *p* = 0.827) or Lysholm (rho − 0.235; *p* = 0.084), (rho − 0.002; *p* = 0.987) (rho − 0.08; *p* = 0.570) score.

**Fig. 2:**
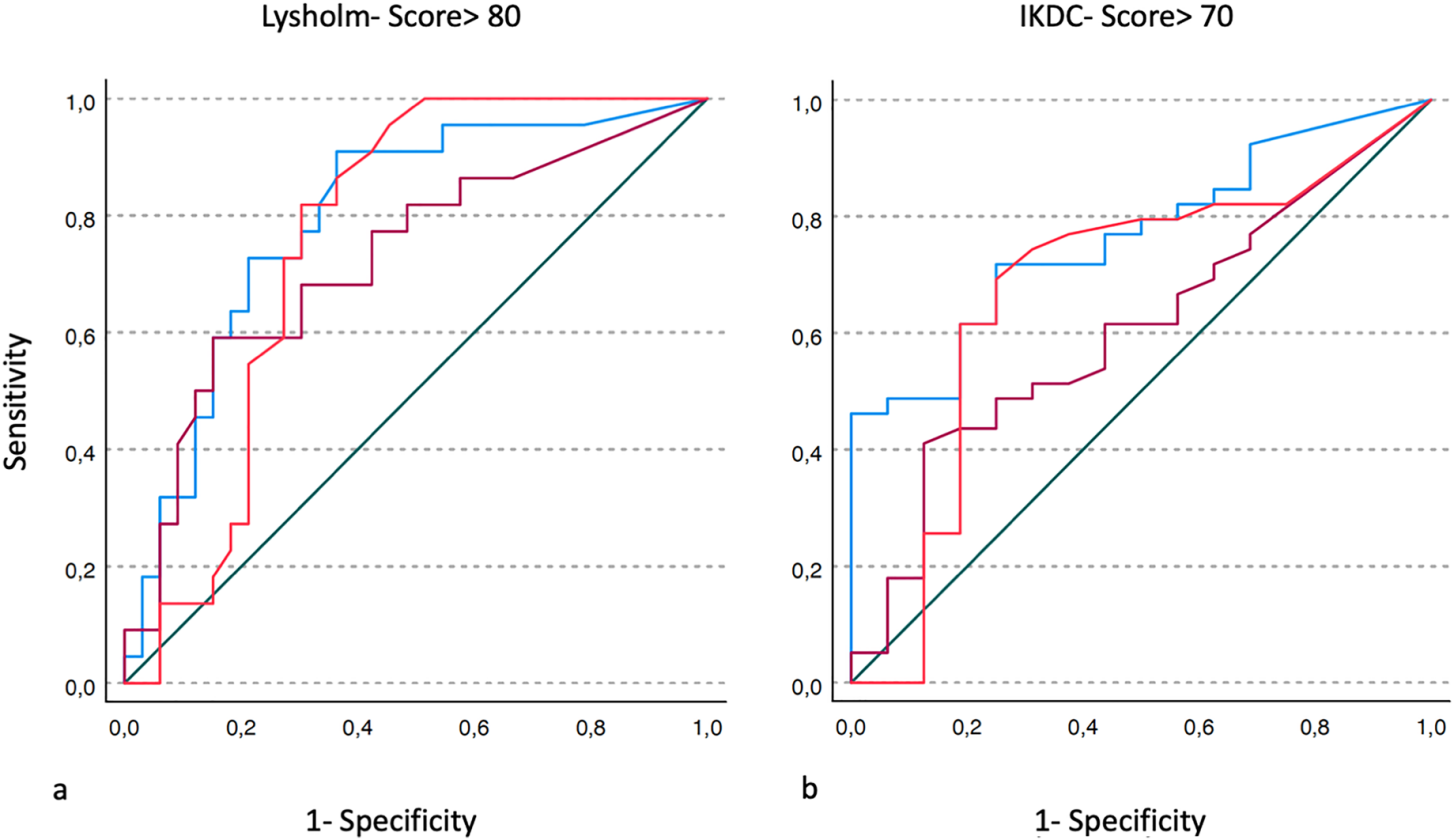
**a** The ROC curves of the step size, gap height and comminution area with regard to a Lysholm score > 80. **b** The ROC curves of the step size, gap height and comminution area with regard to an IKDC score > 70. The “red” line demonstrates the ROC curve of the step height, the “purple” line of the gap size and the “blue” line of the comminution area. The “green” line marks the reference line 0.5

To determine a cutoff value for clinicians to predict acceptable outcomes, we performed ROC analyses of the intraarticular step and gap heights, as well as comminution areas. Figure 2 demonstrates the ROC curves and Table 5 shows the areas under the curve (AUCs) and cutoff values for the intraarticular step and gap heights, as well as for the comminution area.

## Discussion

In our study, we analyzed the outcome of surgical treatment of OTA type B and C tibial plateau fractures as a function of the restoration of articular congruity. Therefore, to the best of our knowledge, we present the largest detailed CT and X-ray analysis of tibial plateau fractures with regard to residual steps, gaps, comminution area and MPTA. The most important finding of our study is the significant correlation of a better outcome with the most well-restored articular surface and joint angle. We could demonstrate that intraarticular steps, gaps, and the size of the comminution area of the tibial plateau influence the clinical outcome. Moreover, we tried to determine the absolute tolerance of articular incongruity in cases of tibial plateau fractures in relation to IKDC- and Lysholm-cutoff values. This is of great clinical significance, since nearly in 30% of all cases intraarticular steps larger than 2 mm in CT scans remain postoperatively with the clinical relevance of these steps remaining largely unknown [22]. Besides a remaining step after reconstruction of the tibial plateau, a gap of the articular surface of the tibial plateau is rarely differentiated. In our study group, the intraarticular step height threshold value to achieve an acceptable outcome score was approximately 2.6–2.9 mm, whereas the cutoff value for gap size was 6.6 mm. Therefore, our results indicate that an intraarticular gap seems to be more tolerable than an intraarticular step, which is supported by a clinical study by Li et al. [10], which also differentiates between step and gap levels. Those authors evaluated the correlation between step size, gap size and clinical outcome in 23 posterior tibial plateau fractures. A significantly negative correlation between residual articular step displacement and clinical outcome was demonstrated, whereas residual gap displacement did not correlate with clinical outcome [10]. Biomechanical findings also support our suggestion since intraarticular gaps led to lower intraarticular friction than intraarticular steps [23].

Furthermore, the severity of the tibial plateau fracture, measured by the number of fracture fragments and the OTA classification, had no significant influence on clinical outcome in our study, which is in line with the results of Freeman et al. [24]. Taken together, the results of our study and those of Freeman et al. suggest that the exact repositioning of the fracture fragments is more decisive for the outcome than the severity itself. Moreover, we were able to confirm the axial alignment, measured as the MPTA, was another major factor influencing the outcome in addition to articular congruity, supporting the idea that the reconstruction of the joint axis is just as important as the reconstruction of the joint surface.

Considering the literature, which addresses the topic of the influence of residual intraarticular step height on functional outcome or development of osteoarthritis after tibial plateau fractures, this shows the inconsistency of the results mentioned above [7, 9, 16–18, 25]. For example, Singleton et al. analyzed the clinical outcome of 41 patients after tibial plateau fracture in terms of articular congruity. Nonoperative as well as operative treated patients were included. The intraarticular step was measured on coronal plane tomograms. Patients were divided into three groups based on the amount of articular depression: < 2.5, 2.5–5.0, and ≥ 5.0 mm. The authors found that patients with an intraarticular step < 2.5 mm had a better functional outcome in terms of range of motion and Oxford, Iowa and Knee Injury and Osteoarthritis Outcome Score (KOOS) scores. In contrast to our study results, the restoration of the mechanical axis did not significantly influence their outcome scores [9]. Furthermore, Parkkinen et al. [7] also tried to identify predictors of early osteoarthritis following lateral tibia plateau fractures as a function of the postoperative mechanical axis and articular congruity. The postoperative radiological evaluation was restricted to standard X-ray images. In summary, a valgus malalignment greater than 5° and an articular depression greater than 2 mm led to advanced osteoarthritis, whereas a normal mechanical axis or a depression less than 2 mm did not. In contrast to our results, clinical outcome was not significantly correlated with the postoperative axis or articular congruity [7]. In addition, Freeman et al. were unable to establish a relationship between clinical outcome and the quality of the reduction [24].

However, there is also literature describing significantly higher levels of articular incongruity being tolerated. Lansinger et al. reviewed the outcomes of 52 patients with tibial plateau fractures and articular depression. Amazingly, comparing residual step-offs of 1–5, 6–10, and more than 10 mm, in their series, “poor” outcomes were only associated with a step-off greater than 10 mm. Patients with a step-off of less than 10 mm achieved “good” or “excellent” outcomes in 96% of cases [15, 18].

As these examples with varying results demonstrate, the answer to the question of the influence of articular congruity on clinical outcome after tibial plateau fracture and the development of osteoarthritis is extremely complex and depends on many factors, such as the interaction of articular congruity, joint stability and axial alignment, which cannot be considered independently. Finally, clinical factors also play an important role in the clinical outcome of tibial plateau fractures, which is demonstrated by the fact that the number of complications and surgical revisions also had a negative impact on the outcome in our study. Therefore, future research is needed on influence- and prognostic factors on the outcome and development of osteoarthritis after tibial plateau fractures.

Despite attempts to ensure reliability, there are certain limitations to our study. First, the retrospective study design led to an inhomogeneous, wide-ranged follow-up period among the patients, which in turn leads to bias in clinical outcome scores. Furthermore, we did not perform long-term radiographic follow-ups as part of the study; therefore, a general statement regarding the postoperative osteoarthritis rate is not possible for all patients. However, the radiological characteristics of osteoarthritis do not seem to be related to lower functional outcomes in the mid- to long term [1]. Furthermore, the restricted inclusion criteria with only analysis of the fractures with existing postsurgical CT lead to the fact that only a subgroup of the tibial plateau fractures is evaluated, which may have led to a certain negative selection in relation to outcome and complications. Nevertheless, our study provides new, valuable results regarding the question of the necessary level of articular congruity of the tibial plateau.

## Conclusion

The most exact possible reconstruction of the articular surface is decisive for the final outcome, since residual intraarticular steps and gaps, a residual comminution area and an axial malalignment in relation to the MPTA lead to reduced activity and outcome scores. Complications and surgical revisions also worsen the postoperative outcome.

## Appendix

See Tables 1, 2, 3, 4 and 5.

**Table 1:** Study group

Study group	
Age (years)	45.5 ± 12.5 (range 17–74)
Sex	
Male	31 (56.4%)
Female	24 (43.6%)
ASA-score	
I	17 (30.9%)
II	32 (58.2%)
III	6 (10.9%)
Comorbidities	
Smokers	7 (12.7%)
Arterial hypertension	9 (16.4%)
Diabetes mellitus	2 (3.6%)
Alcohol abuse	1 (1.8%)
BMI (kg/m^2^)	27.0 ± 6.4 (range,18.4–50.0)
Type of injury	
OTA Type B	27 (49.1%)
OTA Type C	28 (50.9%)
Concomitant injuries	
Bony ACL lesion	3 (5.5%)
Peroneal lesion	1 (1.8%)
Compartment syndrome	9 (16.4%)
Further fractures	37

**Table 2:** Outcome and complications of the study group

IKDC score	62.7 ± 17.6
Lysholm score	80.7 ± 13.3
Pain (NRS)	4.1 ± 2.1
ROM	120° ± 14°
Loss of ROM	10° ± 9° (range, 0°–40°)
Tegner activity score at final follow-up	4*
Tegner activity score before injury	5*
Complications	0.5 ± 0.8 (range, 0–3)
Delayed/impairment of wound healing	15 (27.3%)
Nonunion	5 (9.1%)
Joint/periimplant infection	8 (14.5%)
Intraarticular loose bodies	1 (1.8%)
Screw/material mispositioning	3 (5.5%)
Surgical revisions	0.5 ± 1.1 (range, 0–7)

*NRS* numerical rating scale, *ROM* range of motion

^*^Indicates statistical significance *p* < 0.05

**Table 3:** Postsurgical CT analysis and axial measurement

No. of fragments of the articular surface	2.5 ± 1.2 (1–5)
Step height	3.0 ± 2.2 mm (0–9.3 mm)
Gap size	5.3 ± 4.8 mm (0–20.4 mm)
Articular surface comminution area	586.0 ± 538.6 mm^2^ (0–2551.0 mm^2^)
MPTA	89.9° ± 3.9° (78.9°–102°)
Deviation of the MPTA from the standard (or opposite side)	1.4° ± 2.7° (range, 0°–12°)

**Table 4:** Spearman’s rank correlations between CT measurements and outcome scores

	Step height	Gap size	Comminution area	Deviation MPTA	No. fragments articular surface
Lysholm					
Rho	− 0.3	− 0.3	− 0.4	− 0.3	− 0.2
*p* value	0.012*	0.011*	0.001*	0.032*	0.084
IKDC					
Rho	− 0.3	− 0.3	− 0.4	− 0.3	− 0.2
*p* value	0.037*	0.038*	0.001*	0.039*	0.166

*Statistical significance

**Table 5:** Receiver-operating characteristic (ROC) curves to determine threshold values for the step and gap heights and remaining articular surface destruction

	Lysholm score > 80	IKDC score > 70
Step height		
AUC	0.759	0.675
Threshold	2.9 mm	2.6 mm
Sensitivity	0.818	0.692
1 − specificity	0.303	0.250
*p* value	0.000*	0.045*
Gap size		
AUC	0.725	0.605
Threshold	6.6 mm	6.6 mm
Sensitivity	0.591	0.410
1 − specificity	0.152	0.125
*p* value	0.002*	0.196*
Comminution area		
AUC	0.796	0.765
Threshold	381.00 mm^2^	381.00 mm^2^
Sensitivity	0.909	0.718
1 − specificity	0.364	0.250
*p *value	0.000*	0.000*

*AUC* area under the curve

*Statistical significance
